# Matching X-ray beam and detector properties to protein crystals of different perfection

**DOI:** 10.1107/S1600577514003609

**Published:** 2014-03-18

**Authors:** Colin Nave

**Affiliations:** a Diamond Light Source Ltd, Harwell Science and Innovation Campus, Didcot OX11 0DE, UK

**Keywords:** crystal perfection, synchrotron beam properties, detectors

## Abstract

An analysis is given of the effect of different beam and detector parameters on the sharpness of recorded diffraction features for macromolecular crystals of different quality. The crystal quality parameters include crystal strain, crystal or mosaic block size and mosaic block misorientation. Calculations are given for instrument parameters such as angular resolution of the detector, beam divergence and wavelength bandpass to be matched to the intrinsic diffraction properties from these crystals with the aim of obtaining the best possible data out of each crystal. Examples are given using typical crystal imperfections obtained from the literature for both room-temperature and cryo-cooled crystals. Possible implications for the choice of X-ray source, beamline design, detector specifications, instrument set-up and data processing are discussed, together with the limitations of the approach.

## Abstract 2

Expressions are given to match X-ray data collection facilities to the intrinsic diffraction properties of crystals with different degrees of perfection.

## 1. Introduction   

A primary aim when collecting X-ray diffraction data from radiation-sensitive samples is to obtain the best possible statistics whilst using an X-ray dose sufficiently small to avoid significant damage. This is becoming particularly important with the revival of interest in collecting diffraction data from protein crystals at room temperature and also continuous progress in collecting data from ever smaller crystals at cryo-temperatures. In both cases, the crystals are likely to have a higher degree of perfection than occurs for larger crystals at cryo-temperature. More perfect crystals would mean that beams with lower angular divergence than commonly used could be exploited. This could be particularly important for room-temperature crystals ‘*in situ*’ where increased background could come from the crystallization well (or other container) plus the mother liquor. For very small crystals the ratio of signal to background could also be poor as the total scattering from 1 mm of air is likely to be similar to that given by 1 µm of crystal. In both cases the need to preserve intrinsically sharp diffraction features is important.

This paper covers the implications for setting up the instrument for particular experiments and for designing facilities for collection of data from the most demanding crystals. This includes the specifications of detector systems matched to both the intrinsic properties of the diffraction and the matched beam parameters. The implications for the source are also included. Under the ideal circumstances discussed in this paper, the diffraction data will provide both opportunities and problems for data processing software. The consequences for such software are also discussed.

With low-noise fast-readout detectors the adoption of fine ϕ-slicing as a way of optimizing the spot-to-background ratio is becoming common. Less attention is paid to the size of the diffraction spots at the detector position. The benefits of matching the beam at both the sample position and the detector position can be quite dramatic, as documented by Wikoff *et al.* (2000 ▶). In this case, the virus crystals were large but with a high degree of perfection. An arrangement with a large beam at the sample and a small beam at the detector maximized the spot-to-background ratio while minimizing the radiation damage at the sample.

Adjusting the size and divergence of the incident beam at the specimen position is one way of achieving optimum conditions. In addition, the bandpass of the incident beam can be optimized. If more flux could be obtained by broadening the bandpass then total exposure times will be shorter. This might enable one to outrun radiation damage for non-cryo-cooled crystals (Warkentin *et al.*, 2011 ▶, 2012 ▶; Owen *et al.*, 2012 ▶). However, too broad a bandpass could also degrade the sharpness of the features in the diffraction pattern resulting in a decreased peak-to-background ratio. The importance of minimizing the peak-to-background ratio in order to obtain the optimum data from small crystals has been emphasized by Holton & Frankel (2010 ▶). It is possible that, with a properly matched beam and detector, some of the disadvantages of room-temperature data collection can be compensated for by exploiting the more efficient data collection enabled by retaining the intrinsically sharp diffraction features.

One of the effects of matching the instrument properties to the intrinsic properties of the crystal is that the imperfections of the crystal will be revealed in the diffraction pattern. Such imperfections could, for example, include streaked or split spots. A crystallographer, perhaps used to seeing round spots on other instruments, could conclude that the crystal is unsuitable for data collection. However, it is the premise of this paper that the recording of the details of the diffraction at higher spatial resolution on the detector has the potential to give more accurate intensity measurements, due to a reduction in the area over which the background is recorded. The more detailed information on the spot shape also has the potential to allow enhanced profile fitting.

**Figure 1:**
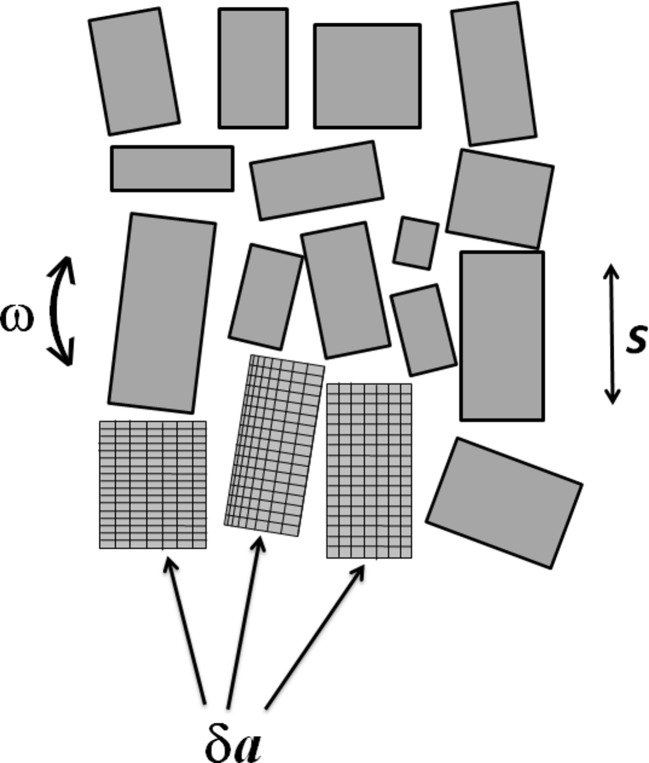
Schematic of the parameters describing crystal imperfections. Both lattice variations between domains and lattice variations within a domain are shown.

In order to keep the analysis general it is kept as simple as possible. The calculations therefore ignore corrections (for example, oblique incidence of the diffracted beam on the detector) for particular diffraction geometries. The nomenclature of Nave (1998 ▶), also adopted by Juers *et al.* (2007 ▶), is used here for the crystal parameters such as crystal/mosaic block size (*s*), angular spread of mosaic block (ω) and strain/variation in cell dimensions (δ*a*/*a*). A modified version of Fig. 1 of Nave (1998) ▶ is shown in Fig. 1 ▶ to illustrate the parameters *s*, ω and δ*a*/*a*. Similar diagrams are provided by Vahedi-Faridi *et al.* (2003 ▶). Although these simple models do not capture some of the details (*e.g.* grain boundaries, dislocations, stacking faults) that may occur in real crystals, the three parameters are sufficient to predict the overall rocking widths and diffracted-beam divergence given by many imperfect crystals.

**Figure 2:**
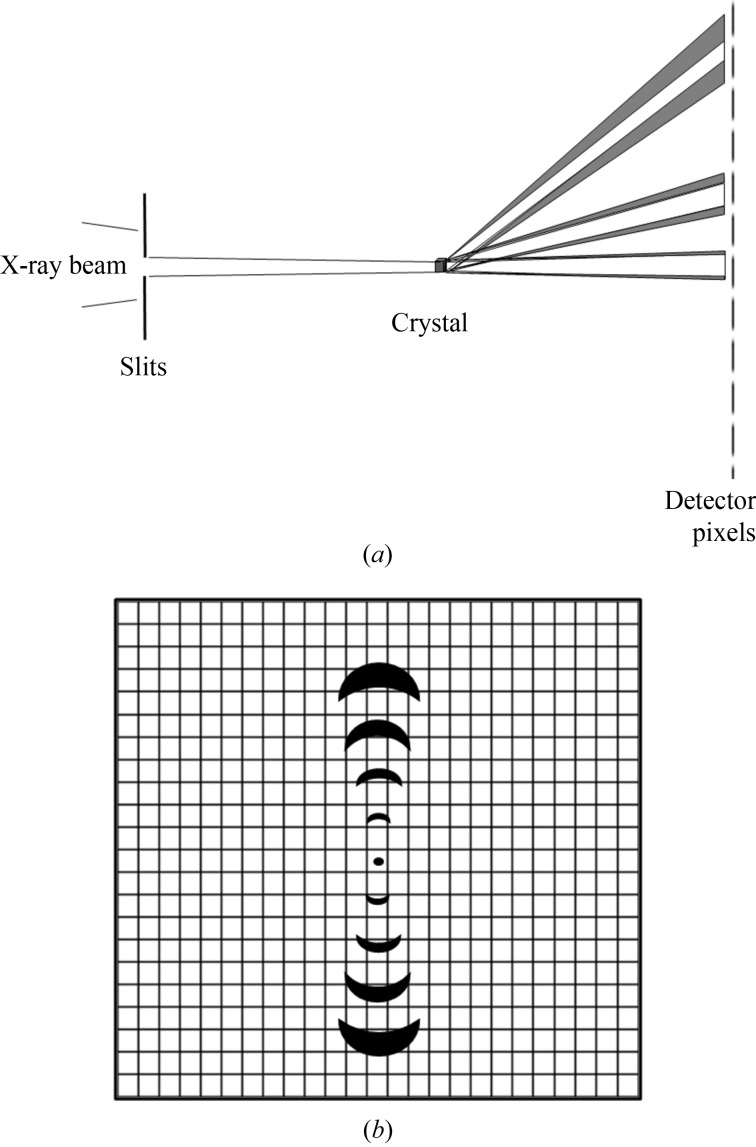
Diagrams showing the effects of sample, beamline and detector parameters on the sharpness of recorded diffraction spots. (*a*) Matching the beam divergence and detector pixels in the presence of lattice variations δ*a*/*a* and finite crystal/domain size *s*. The convergence of the incident X-ray beam is reduced using slits at a distance from the focus. The divergent beam after the crystal is broadened somewhat due to the finite size *s* of the crystal. The broadening of the lower angle reflection is increased further due to the variation in cell dimensions. However, it is reasonably well matched to the detector pixel size when the detector is set at the distance shown. The higher-order reflection at a greater angle is broadened further due to the lattice variation and now occupies several pixels. (*b*) Matching of diffraction spots to the detector resolution in the presence of misorientation of the domains and a variation in the lattice parameter. The first-order reflection occupies one pixel in the azimuthal direction and, by the definition in this paper, is matched to the pixel size when the detector is set at this distance. However, it is not matched in the radial direction. The fourth-order reflection occupies approximately four pixels in the azimuthal direction and one pixel in the radial direction.

Diagrams illustrating the matching between crystal properties, incident beam properties and number of detector pixels are shown in Fig. 2 ▶. In order to show the effects clearly, a detector with a small number of pixels is shown. The various parameters contributing to the beam divergence are correspondingly larger in order to make the effects comparable with the pixel size. As apparent in Fig. 2(*a*) ▶, the diffracted beam size at the detector position can have an additional spatial component given by the size of the crystal (or the size of the beam at the crystal). This effect is not allowed for in the calculations given in this paper but could easily be incorporated.

It is assumed that a match of instrument properties to crystal properties occurs with the same value for spot broadening from each effect. This would mean, for example, an increase in the combined effect of 2^1/2^ assuming the convolution of two equal-width Gaussian functions or a triangular function of twice the full width assuming the convolution of two equal top-hat functions. More or less stringent criteria could be applied. Similar convolutions will occur if several effects (*e.g.* mosaic block size and variation in cell dimensions) are present at the same time (Juers *et al.*, 2007 ▶).

The examples in this paper are for the case of collecting data from macromolecular crystals on a synchrotron source. However, many of the considerations will also apply for other types of crystal and other types of source.

## 2. Expressions for the effect of crystal imperfections on reflection rocking width and diffracted-beam divergence   

The parameters to be matched to the crystal properties are the wavelength bandpass (δλ/λ) of the beam incident on the crystal, the divergence of the incident beam, the rotation range for each image and the number of detector pixels in each direction. If one of these parameters is fixed for practical reasons (*e.g.* the number of detector elements due to cost considerations) then it might be possible to relax other parameters, but the situation would be that, in this example, the incident beam is matched to the detector properties rather than matched to the crystal parameters. The analysis for rocking width and diffracted-beam divergence is carried out assuming a monochromatic beam and a rotating crystal. The analysis for wavelength bandpass is carried out assuming a stationary crystal in a white beam and calculating the wavelength range within a Laue diffraction spot. If a high-quality crystal is rotated in a beam with a broad bandpass, diffraction spots with a radial streak will be obtained, with different parts of the streak corresponding to different Bragg angles. A similar effect will occur with a stationary crystal of lower perfection in a broad-bandpass beam.

Crystal properties vary both from crystal to crystal and within a crystal. For example, the variation in cell dimensions is likely to be different in different directions for crystals with low symmetry. In this case, the beam properties can either be matched to the most demanding direction or, if some compromise is accepted, to some average. As in Nave (1998 ▶) the expressions apply for the case where the reciprocal lattice points pass normally through the sphere of reflection. This will give the minimum rotation range for the reflection and therefore represents the most demanding case.

**Figure 3:**
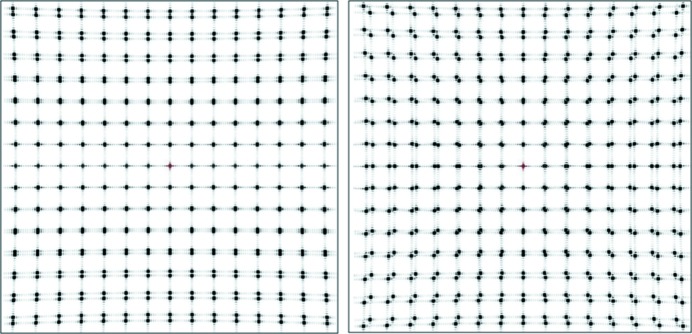
Simulated diffraction patterns from a two-dimensional crystal consisting of two adjacent domains separated in the *b* (vertical) direction. For both diffraction patterns, one domain consists of 10 × 10 lattice points with lattice dimensions *a* = *b*. The second domain consisting of 10 × 10 lattice points with *a*, *b* dimensions (left) *a*, 1.125*a* and (right) 1.125*a*, 1.125*a*. The program *nearBragg* (http://bl831.als.lbl.gov/~jamesh/nearBragg/) from James Holton was used for this calculation.

In the general case, a variation in cell dimensions produces spots which are elongated in the radial direction as well as in the azimuthal direction. However, the spot shape will depend on the Miller indices of the reflection as well as the variation of the cell dimensions in each direction. To illustrate this, two-dimensional simulations are shown in Fig. 3 ▶, with two different lattice parameters existing in the same crystal. The simulation (left) with a lattice difference in just the *b* (vertical) direction shows constant spot width horizontally and splitting in the vertical direction. The simulation (right) with lattice differences in both the *a* and *b* directions shows radially extended splitting. The splitting of the spots at intermediate resolutions is similar to that shown in Fig. 3 of Nave (1998 ▶). A continuous lattice variation would replace the split spots with a continuous streak in the same direction as the splitting. A paper on measurement of spot shapes using a coherent beam and a high-resolution detector is in preparation (Nave *et al.*, 2014 ▶).

Table 1 ▶ gives expressions for the effect of various crystal imperfections on reflection rocking width and diffracted-beam divergence. It is assumed for this purpose that the crystal imperfections are isotropic. The analysis for diffracted-beam divergences and crystal rocking widths in a monochromatic beam follows Nave (1998 ▶) and Juers *et al.* (2007 ▶). A distinction can be made between the variation in cell dimension within a mosaic block (*e.g.* due to elastic strain) and cell variations between blocks. In practice the effects on the broadening of diffraction features is similar (Stokes & Wilson, 1944 ▶) and they are treated as the same in the calculations given here. The distinction is important if interested in the crystal properties or for separating out regions of the crystal with different unit-cell parameters (see §4.5). For a beam with a broad bandpass, the analysis of Laue diffraction spot shapes can be used. The expression for the azimuthal divergence of a spot produced by a mosaic crystal is 2ω sin(θ) and the radial divergence in a white beam is 2ω (Ren *et al.*, 1999 ▶).

The analysis of matching the beam divergence to the crystal rocking width is dependent on the angle at which the reflection crosses the Ewald sphere. For the case where the rotation axis is horizontal and normal to the incident beam direction and the reflection occurs at right angles to the rotation axis, the vertical beam divergence is the relevant one for matching to the rocking width of the reflection. For this configuration, the vertical beam divergence is also relevant to matching the radial divergence of the diffracted beam. Again for this geometry, the horizontal beam divergence is matched to the azimuthal spread of the diffracted beam (*e.g.* for the arcs produced by a crystal with a distribution of angles between mosaic blocks).

**Figure 4:**
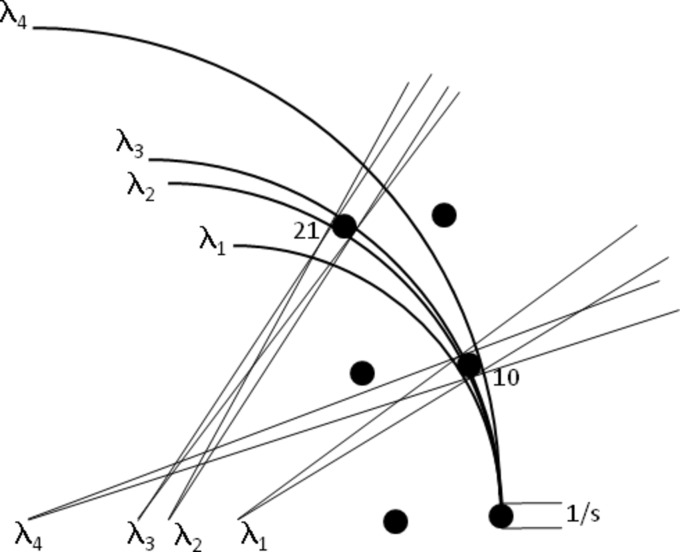
Matching of wavelength bandpass to the diffraction properties for a crystal or mosaic block of size *s*. The bandpass is matched to the rotation range for the 10 reflection but an increase in the diffracted-beam divergence is present. The diffracted-beam divergence for the 21 reflection is similar to that given by a monochromatic beam but only a portion of the wavelength range is contributing to the reflection.

As an example of the matching of wavelength bandpass, Fig. 4 ▶ shows diffraction spots which are enlarged due to a finite crystal/mosaic block size. The 10 reflection is (by definition) matched to the wavelength bandpass as all wavelengths are contributing to the diffraction. However, increased divergence and enlarged diffraction spots on the detector will occur compared with a strictly monochromatic situation. The bandpass is therefore matched to rocking width for this reflection but not to the diffracted-beam divergence. In the case of the 21 reflection, only a portion of the wavelengths are contributing. For this case, the divergence (angle between λ_2_ and λ_3_ at the reciprocal lattice point) produced by the wavelengths which do contribute is matched to the divergence (angle subtended by the broadened reciprocal lattice point at λ_2_ or λ_3_) produced when the broad reflection crosses a strictly monochromatic beam. However, the additional wavelengths between λ_1_ and λ_4_ still contribute to the background. The match between δλ/λ and the diffracted-beam divergence is calculated as follows:

Wavelength range contributing: δλ/λ = δθ cot θ.

Divergence for strictly monochromatic beam: 2δθ = λ/*s*.

Matching: δλ/λ = λ cot(θ)/(2*s*).

The expressions for matching the number of detector elements from the detector centre is given by the diffraction angle [2θ = 2 sin^−1^(λ/2*d*)] divided by the angular spread of the diffracted beam. A calculation of the number of detector elements required to preserve the sharp features assumes that the intrinsic width of the diffraction feature occupies one detector resolution element. This would typically be a single pixel for a pixel detector or the width of the point-spread function for a CCD- or image-plate-based system. It should be noted that this matching would give a significant limitation for profile fitting in the case of a pixel detector (see §4.5).

Where there is a finite value of ω, any wavelength bandpass in the beam will produce a finite beam divergence in the radial direction, thereby degrading the signal-to-background ratio for the intrinsically sharp diffraction arc. Figures for matching the wavelength bandpass with the instrinsic width of the diffraction are therefore not provided for this case.

For a flat detector at right angles to the incident beam, two additional terms are necessary in order to derive the size of a diffraction spot on the detector. Firstly, a *D*/cos(2θ) term takes account of the distance travelled by the diffracted beam to the detector, where *D* is the crystal-to-detector distance along the incident beam direction. An additional 1/cos(2θ) term applies in the radial direction and allows for the non-normal incidence as a function of diffraction angle. In order to keep the expressions general, these corrections are not applied in the expressions in Table 1 ▶ and the calculations given in Tables 2–5.

## 3. Examples of matching the crystal parameters to the beam and detector characteristics   

For the examples below, a wavelength of 1 Å is assumed. Values for various crystal imperfections are taken from the literature and values are then chosen to represent protein crystals at room temperature and cryo-temperature.

In order to keep the calculations realistic, only two significant figures are given in Tables 2–5. It is, however, worth noting that in some cases this hides the small differences between the numbers. For example, values of 250 are given for the number of detector elements in the last two columns of Table 4. More precise values are 251 and 253. The difference reflects the non-linear relationship between 1/*d* and 2θ which would be significant for data at higher resolution or obtained at longer wavelength.

### 3.1. Matching properties to a perfect crystal (or mosaic blocks) of size *s*   

Estimates of mosaic block sizes for crystals at room and cryo-temperature can be determined by measuring the rocking width of reflections as a function of resolution. Values for cryo-cooled crystals include 0.25 µm (Leslie *et al.*, 2012 ▶) and 0.4 µm (Juers *et al.*, 2007 ▶). At room temperature, much larger mosaic block sizes can occur, *e.g.* >10 µm (Juers *et al.*, 2007 ▶). For microcrystals, Holton & Frankel (2010 ▶) have discussed the minimum crystal size needed to collect a complete data set and derived values between 0.34 µm and 1.2 µm dependent on various assumptions. In order to represent the effects of a variation in crystal or mosaic block sizes, values of 0.5 µm and 10 µm are used for the calculations in Table 2 ▶.

### 3.2. Matching properties to a mosaic crystal with large mosaic blocks and mosaicity (misorientation parameter) ω   

For this case, the effects on the diffraction pattern in the radial and azimuthal directions are quite different. In a strictly monochromatic beam, sharp arcs are present with an angular width in the azimuthal direction corresponding to the mis­orientation parameter. The beam divergence and number of detector elements are calculated to match this. The calculations in Table 3 ▶ are carried out for mosaicities of 10^−4^ rad (0.0057°) and 4 × 10^−3^ rad (0.228°), representing typical values found for crystals at room temperature and large crystals at cryo-temperature (*e.g.* Vahedi-Faridi *et al.*, 2003 ▶).

### 3.3. Matching properties to a crystal with strain δ*a*/*a*   

A variation in cell dimensions produces a similar effect on the reflection rocking widths to an angular distribution of mosaic blocks. However, the effect on the diffracted-beam divergence is different. A variation in cell dimensions produces spots which are elongated in the radial direction as well as the azimuthal direction. By examining the spot shapes it appears that in many cases the variation in cell dimensions is the dominant factor for many cryo-cooled crystals (Nave, 1998 ▶; Juers *et al.*, 2007 ▶; Diederichs, 2009 ▶) although effects due to misorientation between mosaic blocks also occur (*e.g.* Juers *et al.*, 2007 ▶). Variations in cell dimensions δ*a*/*a* of 0.005–0.015 (Nave, 1998 ▶) and 0.0038 (Juers *et al.*, 2007 ▶) have been estimated for cryo-cooled crystals. The lower values of reflection rocking widths for crystals at room temperature (*e.g.* Vahedi-Faridi *et al.*, 2003 ▶) are consistent with a variation in cell dimensions of 10^−4^ or less. Values for δ*a*/*a* of 4 × 10^−3^ and 10^−4^ are used in Table 4 ▶ to represent crystals at cryo-temperature and room temperature.

### 3.4. Summary of matching for crystals at room temperature and cryo-temperature   

Calculations are given in Table 5 ▶ for matching the instrument properties to typical crystals at room temperature and cryo-temperature. It is assumed that the various effects give Gaussian distributions for the three-dimensional diffraction profile and that these Gaussians can be combined in quadrature. However, for these examples, the effects of the variation in cell dimensions dominate compared with the effects due to the finite mosaic block size. A narrower bandpass is required to match the diffracted-beam divergence compared with that required to match the rocking width. A bandpass of 1.1 × 10^−4^ for the room-temperature crystal and 4 × 10^−3^ for the crystal at cryo-temperature would therefore be matched to the crystal properties.

Present detectors do not have the number of resolution elements (*e.g.* pixels) matched to the small diffracted-beam divergences for the crystal at room temperature. The last two rows in the table therefore give values for the incident beam divergence and wavelength bandpass matched to a detector with 1000 resolution elements from the centre of the diffraction pattern to the resolution limit. This corresponds to 2000 × 2000 elements for a centred detector.

## 4. Implications of matching the crystal and instrument properties   

Matching of the instrument properties to the crystal properties will have implications in several areas as described in this section

### 4.1. Implications for X-ray source   

The combination of crystal size and matching incident beam divergence can be used to define the beam acceptance of the crystal and the emittance matching of the source. For the example at room temperature shown in Table 5 ▶, a beam emittance of 5.4 nm rad (FWHM values) into a bandpass of 1.1 × 10^−4^ provides a match to the crystal properties. A source which maximizes the number of photons within this emittance volume would therefore be matched to the crystal properties and provide fast data collection.

If the crystal has a high degree of perfection, then emittances (*e.g.* 0.1 nm rad) approaching those for a fully coherent beam would provide a match to the crystal properties. The aim would be to match the incident beam divergence to the crystal rather than to the domain size. A beam with a high coherence would also be required if it is intended to use the continuous X-ray diffraction approach (Dilanian *et al.*, 2013 ▶; Elser, 2013 ▶) for analysis of the diffraction pattern. A high coherent flux is obtained by maximizing the spectral brightness of the source, allowing for the issue (Mills *et al.*, 2005 ▶) that the normal units for this quantity are photons s^−1^ mm^−2^ mrad^−2^ (0.1% bandpass)^−1^ and that the emittance and bandpass parameters in some of the examples given here are significantly smaller than these units.

Both free-electron lasers and the latest synchrotron storage ring and energy-recovery linac designs have these small emittances albeit into a bandpass larger than 10^−4^. However, provided appropriate monochromator and collimation arrangements are incorporated into the beamline design, a matching can be achieved on virtually any source if a loss of flux is accepted. This approach was used by Nave (1998 ▶) in conjunction with a bending magnet rather than a much lower emittance undulator source. The loss of flux will affect the sample throughput but this might be considered acceptable given the short exposure times and crystal lifetime on many synchrotron sources. However, the loss of flux would affect the possibility of outrunning radiation damage for room-temperature data collection.

The minimum crystal size for collecting data from a crystal at cryo-temperature has been discussed by Holton & Frankel (2010 ▶). A free-electron laser source can provide X-rays with sufficiently short pulses to circumvent radiation damage for very small crystals (Barty *et al.*, 2012 ▶). The maximum number of photons per pulse from these sources provides a limitation for data collection from larger crystals. For these crystals, it would be possible to have more incident photons with a continuous source before radiation damage occurs at cryo-temperature. There will be a crossover point for crystal size [see the discussion by Cowan & Nave (2008 ▶)] where a free-electron laser source will provide more information than a continuous source. However, in both cases an optimized set-up will be required to match the intrinsic properties of the crystal diffraction and achieve the full potential of these sources.

### 4.2. Implications for beamline design   

The desire to have a sub-micrometre focus (implying strong demagnification) conflicts somewhat with the desire to have a small beam divergence. The beam divergences can be reduced relatively easily with the use of slits some distance from the focus (see Fig. 2 ▶) with the consequent reduction in flux. Alternatively, the beam could be focused on the detector rather than the sample. As the product of beam size and divergence will be conserved, the arrangement which is simplest to achieve is likely to be chosen.

For the examples given here, the value of δλ/λ necessary to preserve the diffracted-beam divergence is less than that needed to preserve a narrow rocking width. However, the required number of detector elements is approximately the inverse of the value of δλ/λ. For the most perfect crystals, the required number of detector elements can be very large. This is likely to be the limiting factor in many cases so some relaxation of the wavelength bandpass and incident beam divergence would be reasonable until detectors with more resolution elements become available.

The bandpass matching for the room-temperature crystal in Table 5 ▶ is similar to the bandpass of 1.2 × 10^−4^ for the commonly employed Si(111) monochromator and broader than that given by higher-order reflections from silicon monochromators. For less perfect sample crystals (*e.g.* at cryo-temperature) a broader bandpass and increased flux can be achieved by varying crystal monochromator parameters such as asymmetric cut and crystal composition (*e.g.* germanium rather than silicon). Multilayer monochromators can provide a bandpass of approximately 0.4% at 12 keV with a 50% peak reflectivity (Oberta *et al.*, 2012 ▶). An instrument equipped with both multilayer and single-crystal monochromators is worth considering. This could match a wide range of crystal types with the disadvantage of some increased complexity and cost.

The main implication for beamline design is to have enough flexibility in the beam collimation, focusing optics and ideally monochromatization to match a wide range of specimens.

### 4.3. Implications for detector specifications   

For many cases, present detectors have fewer resolution elements than indicated in Tables 2 ▶–5 ▶. This is probably the major limitation of present instruments. The matching of a detector pixel size to the size of a diffraction feature (as in this paper) is itself somewhat of a compromise because the intensity can vary in a non-linear fashion across a pixel. This would occur for example if the pixel size was the same as the FWHM of a Gaussian-shaped spot and the pixels were independent (as in the case of a pixel detector but not in the case of an indirectly illuminated CCD detector). A non-linear variation of intensity across a pixel could affect profile fitting (see §4.5).

### 4.4. Implications for instrument set-up   

For some conditions, the matching of beam properties to crystal properties is only weakly dependent on the resolution. An example is the matching of wavelength bandpass to diffracted-beam divergences (see Table 4 ▶) for the case of a crystal with lattice strain. In this case, the number of detector elements is also only weakly dependent on the resolution. This is because the increase in spot size at high resolution compensates for the increase in 2θ. In other cases, the matching is dependent on the resolution, for example matching the wavelength bandpass to the crystal or mosaic block size (see Table 2 ▶). In these latter cases, the parameters should be optimized for the weaker data at high resolution where the background will be the limiting factor.

With a flat detector, detector distances will differ from the centre to the outside. For most cases, one would presumably optimize things for the weaker spots near the detector edges. The optimum would be a curved detector with the sample and detector on a Roland circle.

Further analysis could be carried out using the ray-tracing approach of Diederichs (2009 ▶) or Schreurs *et al.* (2010 ▶) for the part related to the protein crystal. In principle this could be combined with ray tracing for the X-ray optics. An alternative is to include the crystal as an optical element in the beamline and extend phase-space analysis (*e.g.* Ferrero *et al.*, 2008 ▶) to examine the properties of the diffracted beams at the detector. These methods are based on Gaussian, Lorentzian or top-hat functions for the various parameters. In some cases, a small number of domains are present in the crystal, giving structured diffraction features corresponding to blocks with different discrete orientations or unit-cell parameters. In order to preserve this information, the instrument characteristics should ideally be matched to these individual sharp features in the diffraction pattern. In some cases it is possible to separate out the individual domains by two-dimensional raster scanning using a small X-ray beam, allowing the best diffracting region to be selected (*e.g.* Bowler *et al.*, 2010 ▶). If this is not possible (*e.g.* because the domains are distributed throughout the three-dimensional volume of the crystal), different domains can still be separated provided the beam characteristics are matched to the intrinsic width of the diffraction features. The presence of complex spot profiles (and coherence effects) will require additional procedures for data analysis (see §4.5).

In Table 5 ▶ the matching of the beam divergence and bandpass to a detector with 2000 × 2000 elements is given. If these parameters are matched to the detector rather than the crystal properties, an increased bandpass and beam divergence is calculated for the room-temperature crystal. However, the intrinsic rocking width of the crystal would be degraded by such an increase in bandpass and divergence of the incident beam so the smaller values for these parameters would still give an advantage. For the crystal at cryo-temperature, the 2000 × 2000 element detector might be considered to be over-specified. However, the matching discussed in this paper does still result in significant degradation and under sampling of the diffraction pattern. The diffraction spots on the 2000 × 2000 element detector would occupy approximately four pixels, allowing reasonable sampling of the diffraction profiles.

If a crystal has one large unit-cell dimension, it is common practise to try to align this along the rotation axis. This means that larger rotation increments can be collected before spot overlap occurs. With a diffraction set-up matched to the intrinsic properties of the crystal, this type of procedure would not be required as the three-dimensional profile of each spot is fully determined. The matching advocated here would give the maximum chance of deconvoluting spots which, due to the crystal rather than instrument properties, are intrinsically overlapping.

For larger crystals, it is common to use multi-pass data collection and helical scans. Alternatively, the rotation axis can be offset from the centre of the crystal. These arrangements are used to mitigate the radiation damage by spreading a small beam over a much larger crystal. If the crystal is of uniform quality, it would often be sensible to use a beam matched to the crystal size. If this is achieved by altering the focusing, then a less divergent beam would result, perhaps leading to a better matching with the intrinsic properties of the crystal diffraction. For many anomalous-dispersion studies, multi-pass data collection would still be valuable as it is advantageous to compare related reflections which have received a similar dose. Helical scans can achieve this if, for example, a full 360° of data are collected at one wavelength and the next wavelength collected from a fresh length of crystal, each part of which receives a similar dose to the previous wavelength at the same orientation.

### 4.5. Implications for data processing   

Leslie *et al.* (2012 ▶) included effects of small mosaic block size to obtain a better expression for the variation in the angular range of reflections as a function of resolution, estimating a mosaic block size of 0.2 µm for the protein crystal examined. It is possible that this type of analysis could be extended by using the expressions for reflection rocking widths and diffracted-beam divergences. This would be done by exploiting the relationships between the predicted spot profiles at different resolutions. These spot profiles are also linked to the reflection rocking width at different resolutions. Recording the full three-dimensional profiles of the reflections would be necessary to refine all the parameters of the model. Software for doing this is being developed (Schreurs *et al.*, 2010 ▶). This approach should be useful if the reflection profiles are relatively simple, for example consisting of a limited number of overlapping Gaussians. If this is the case, the relationships could be used to assist in profile fitting and reflection rocking width determination. In simple terms, if information is present on the three-dimensional profiles of the (*hkl*) 100, 200 and 300 reflections it should be possible to predict the profiles of higher orders. This type of analysis would also provide values for the parameters describing crystal imperfections enabling more information about these parameters to be obtained for crystals at room temperature and cryo-temperature.

If there are a small number of individual domains which can be resolved with a high-resolution instrument, it provides the possibility of processing the data separately from each of these domains. Separate domains could have different structures especially if they have different unit-cell parameters. Refining these domains independently might lead to better structural models [for a discussion on the possibilities, see Pozharski (2012 ▶)].

**Figure 5:**
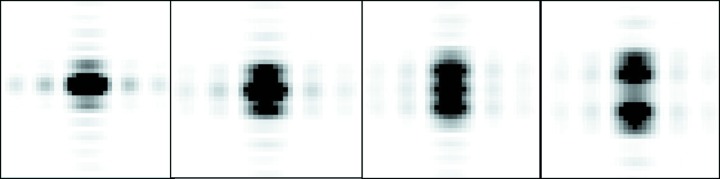
Magnified images of the *hk* 01, 02, 03 and 04 simulated diffraction spots in Fig. 3 ▶ showing the interference effects between the two domains.

If the beam on the crystal has a high degree of coherence and there are a limited number of domains, interference effects between domains could be present on the diffraction pattern. This is illustrated in Fig. 5 ▶. The interference effects are similar to those produced by two slits with a separation comparable with the slit widths. The shape of the features indicates that the crystal is twice as long in the vertical direction as in the horizontal direction. For this fully coherent case, it would be necessary to analyse the different interference functions around each spot in order to predict the spot shape around higher-order spots. For a large number of mosaic blocks with, for example, a Gaussian distribution of displacements, misorientations and unit-cell parameters, the interference effects would average out to give the simpler matching to the instrument parameters. However, the possibility of observing interference effects between mosaic blocks should not be ignored as it gives both problems and opportunities for data processing. It is also worth pointing out that coherent illumination of the crystal will give speckle in the background. Measurement of this speckle would provide more information than that provided by normal diffuse scattering. However, the presence of the speckle would complicate background subtraction when determining the integrated intensity of diffraction spots.

It should also be possible to analyse the peak shapes of the type shown in Fig. 5 ▶ using the continuous diffractive field (*e.g.* Dilanian *et al.*, 2013 ▶; Elser, 2013 ▶). The sampling required by the detector for such coherent diffractive imaging measurements is a factor of two finer than that required for matching the angular resolution of a detector pixel to the beam divergence. This type of analysis can, at least in principle, produce a domain model of the crystal which could be exploited for further analysis. Interference effects within the crystal can also be modelled by a spatial variation in real space phase and such methods have been used for modelling the diffraction from domains within twinned crystals (Aranda *et al.*, 2010 ▶).

The case of partially coherent illumination of the crystal is more complex. The matching of beam divergence to the size of mosaic blocks in a crystal means that each mosaic block is coherently illuminated by the incident beam but the crystal as a whole is not coherently illuminated. Interference effects between blocks which are coherently illuminated together could occur but this interference will not extend throughout the whole crystal. Methods for analysing diffraction from partially coherent beams are being developed (*e.g.* Whitehead *et al.*, 2009 ▶). The issue is raised here as a possible complication for analysing spot profiles.

When profile fitting, the intensity recorded for a pixel is assumed to be located at the centre of the pixel. A scale factor is then obtained between the observed and a known (learned) profile measured across several pixels. The scale factor is then used to calculate the intensity of the spot. In order for profile fitting to be successful, the detector has to sample the profile at a sufficiently fine interval. Indirectly coupled CCD detectors have a broad point-spread function due to limitations in the phosphor and fibre optics. This is a disadvantage as it means that the spots are spread out over many pixels. It does, however, make any profile fitting in *x* and *y* easier as the pixels will give reasonably adequate sampling of the broadened reflection profiles. For the pixel detectors, the individual pixels are much more independent. Most of the intensity of a diffraction spot can fall within one pixel with much weaker values in neighbouring pixels. For these detectors, a pixel does not sample the image but averages (*i.e.* as in a histogram) the values over the area of the pixel. This average will only correspond to the value at the centre of the pixel if the intensity varies linearly across the pixel (similar arguments apply for the sampling in the ϕ direction). If the linearity condition is not met then a discrepancy will be seen when comparing diffraction spots with intrinsically identical profiles. The magnitude of this discrepancy will depend on the extent to which the intensity varies in a non-linear manner across a pixel or throughout the rotation range for an image. Fig. 2 ▶ shows reflections where the linear condition does not hold and errors would be expected to occur when profile fitting.

One possible solution to the problem of pixel sampling (other than having many more detector pixels to ensure a linear change of intensity across each pixel) is to have separate models of the spot profiles and the detector. As the spot profiles are refined, they can be mapped to the detector in a manner which properly addresses the detector histogramming at each pixel. This would by-pass the sampling problem at the cost of increased complexity. Several diffraction spots would be required to obtain reliable profiles. These profiles could be determined in reciprocal-space coordinates rather than in detector coordinates and then mapped to the detector coordinates. Such a procedure appears attractive as it should be possible to derive a single model for the spot profiles which can be applied to all the diffraction spots. The problem could then be well determined, despite the under-sampling of individual diffraction spot profiles. One issue will be incorporating changes to the spot profiles due to radiation damage.

If operating with broader bandpass beams and high-quality crystals, sharp spots will still occur if the crystal is not rotated. This would give the most favourable spot-to-background ratio. One issue will be whether monochromatic or Laue software packages will give the best results when processing such images.

## 5. Conclusions   

If the crystals have a degree of perfection similar to high-quality room-temperature crystals, then the beam divergences and X-ray bandpass will have to be kept small and detectors with good angular resolution will have to be available if one aims to preserve the intrinsically sharp diffraction features. This will optimize the recording of weak data above a high background. Similar instrument properties will also benefit the recording of data from less perfect crystals, if the diffraction from these crystals includes intrinsically sharp features (*e.g.* due to a small number of domains or due to coherence effects). Additional procedures will be required in the data analysis software in order to develop and exploit a model of such features.

The analytical expressions in this paper should provide an understanding of how to optimize the data collection facilities for particular cases. In particular they illustrate that the presently available detectors are not fully matched to the intrinsic diffraction properties from many crystals. The numbers calculated for the number of detector elements to match the diffraction features are, if anything, rather conservative as they do not take into account the optimum sampling of diffraction profiles, especially with pixel detectors. Advances in area detector technology (*e.g.* film, proportional chambers, image plates, CCD detectors, pixel detectors) have always resulted in significant improvements in the quality of X-ray diffraction data. This paper predicts that this will continue.

## Figures and Tables

**Table 1:** Expressions for the effect of crystal imperfections on reflection rocking width and diffracted-beam divergence

	Diffracted-beam divergence	Rocking width	Number of detector resolution elements from centre	δλ/λ matched to rocking width	δλ/λ matched to diffracted-beam divergence
Mosaic block (or sample) size, *s*	λ/*s*	*d*/*s*	2θ *s*/λ	*d* cot(θ)/*s*	λ cot(θ)/(2*s*)
Angular spread of blocks, ω	Azimuthal arcs width 2ω sin(θ)	ω	2θ/[2ω sin(θ)]	ω cot(θ)	0 (not applicable)
Variation in cell dimension, δ*a*/*a*	(λ/*d*)(δ*a*/*a*)	δ*a*/*a*	2θ/[(δ*a*/*a*)(λ/*d*)]	cot(θ) δ*a*/*a*	(λ/2*d*)(δ*a*/*a*) cot(θ)

**Table 2:** Matching properties to a perfect crystal (or mosaic blocks) of size *s*

	10 µm crystal, 5 Å resolution	10 µm crystal, 2 Å resolution	0.5 µm crystal, 5 Å resolution	0.5 µm crystal, 2 Å resolution
Beam divergence (rad) [°]	10^−5^ [0.00057]	10^−5^ [0.00057]	2 × 10^−4^ [0.011]	2 × 10^−4^ [0.011]
Rotation range (rad) [°]	5 × 10^−5^ [0.0028]	2 × 10^−5^ [0.0011]	10^−3^ [0.057]	0.4 × 10^−3^ [0.023]
Number of detector elements from centre	2.0 × 10^4^	5.0 × 10^4^	1 × 10^3^	2.5 × 10^3^
δλ/λ matched to rocking width	5.0 × 10^−4^	7.7 × 10^−5^	9.9 × 10^−3^	1.5 × 10^−3^
δλ/λ matched to diffracted-beam divergence	5.0 × 10^−5^	1.9 × 10^−6^	9.9 × 10^−4^	3.9 × 10^−4^

**Table 3:** Matching properties to a mosaic crystal with large mosaic blocks and misorientation parameter ω The beam divergence and number of detector elements are calculated to match the spread of the spots in the azimuthal direction.

	ω = 10^−4^, 5 Å resolution	ω = 10^−4^, 2 Å resolution	ω = 4 × 10^−3^, 5 Å resolution	ω = 4 × 10^−3^, 2 Å resolution
Beam divergence (rad) [°]	10^−4^ [0.0057]	10^−4^ [0.0057]	4 × 10^−3^ [0.23]	4 × 10^−3^ [0.23]
Rotation range (rad) [°]	10^−4^ [0.0057]	10^−4^ [0.0057]	4 × 10^−3^ [0.23]	4 × 10^−3^ [0.23]
Number of detector elements from centre	10000	10000	250	250
δλ/λ matched to rocking width	9.9 × 10^−4^	3.9 × 10^−4^	4.0 × 10^−2^	1.5 × 10^−2^

**Table 4:** Matching properties to a crystal with strain δ*a*/*a*

	δ*a*/*a* = 10^−4^, 5 Å resolution	δ*a*/*a* = 10^−4^, 2 Å resolution	δ*a*/*a* = 4 × 10^−3^, 5 Å resolution	δ*a*/*a* = 4 × 10^−3^, 2 Å resolution
Beam divergence (rad) [°]	2 × 10^−5^ [0.0011]	5 × 10^−5^ [0.0028]	8 × 10^−4^ [0.046]	2 × 10^−3^ [0.11]
Rotation range (rad) [°]	10^−4^ [0.0057]	10^−4^ [0.0057]	4 × 10^−3^ [0.23]	4 × 10^−3^ [0.23]
No of detector elements from centre	1.00 × 10^4^	1.0 × 10^4^	2.5 × 10^2^	2.5 × 10^2^
δλ/λ matched to rocking width	9.9 × 10^−4^	3.9 × 10^−4^	4.0 × 10^−2^	1.5 × 10^−2^
δλ/λ matched to diffracted-beam divergence	9.9 × 10^−5^	9.7 × 10^−5^	4.0 × 10^−3^	3.9 × 10^−3^

**Table 5:** Examples from cryo- and room-temperature crystals at 2 Å resolution Room temperature: crystal size 100 µm, mosaic block size 5 µm, δ*a*/*a* = 10^−4^. Cryo-temperature: crystal size 20 µm, mosaic block size 0.5 µm, δ*a*/*a* = 4 × 10^−3^. The last two rows refer to a detector with 1000 elements from the centre.

	Room temperature	Cryo-temperature
Beam divergence (rad) [°]	5.4 × 10^−5^ [0.003]	2 × 10^−3^ [0.11]
Rotation range (rad) [°]	1.1 × 10^−4^ [0.006]	4 × 10^−3^ [0.23]
Number of detector elements from centre	9.3 × 10^3^	250
δλ/λ matched to rocking width	4.2 × 10^−4^	1.5 × 10^−2^
δλ/λ matched to diffracted-beam divergence	1.1 × 10^−4^	4 × 10^−3^
Beam size at sample (µm)	100	20
Beam emittance (nm rad) (FWHM values)	5.4	40
Beam divergence matched to detector	5 × 10^−4^	5 × 10^−4^
δλ/λ matched to detector	10^−3^	10^−3^
